# Supplementary material for: SARS-CoV-2-specific T cell memory is sustained in COVID-19 convalescent patients for 10 months with successful development of stem cell-like memory T cells
Source: Nat Commun. 2021 Jun 30;12:4043. doi: 10.1038/s41467-021-24377-1 (PMC8245549; doi:10.1038/s41467-021-24377-1)
Supplement: Supplementary file 1 — Supplementary Information [file 41467_2021_24377_MOESM1_ESM.pdf]

SARS-CoV-2-specific T Cell Memory is Sustained in COVID-19 Convalescent Patients for 10 Months with Successful Development of Stem Cell-like Memory T Cells

Jae Hyung Jung[†], Min-Seok Rha[†], Moa Sa, Hee Kyoung Choi, Ji Hoon Jeon, Hyeri Seok, Dae Won Park, Su-Hyung Park, Hye Won Jeong*, Won Suk Choi*, Eui-Cheol Shin*

Supplementary Information

Supplementary Figure 1

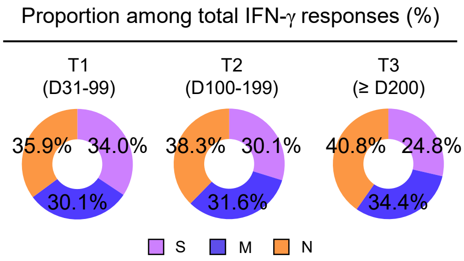

Supplementary Figure 1. Proportion of S-, M-, and N-specific IFN-γ responses among total IFN-γ responses. PBMC samples from COVID-19 convalescent patients were stimulated with OLPs of S, M, or N (1 μg/mL) for 24 h and spot-forming units of IFN-γ-secreting cells were examined by ELISpot. Pie charts showing the proportion of S-, M-, and N-specific IFN-γ responses among the total IFN-γ responses in T1 (n=49, 31 - 99 DPSO), T2 (n=41, 100 - 199 DPSO), and T3 (n=31, ≥ 200 DPSO).

Supplementary Figure 2

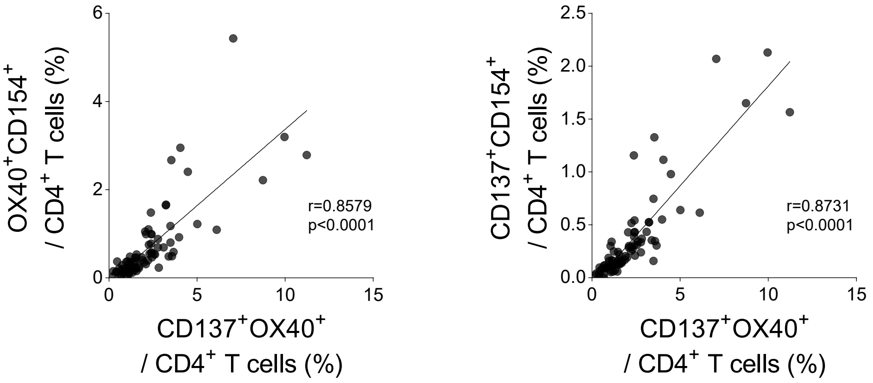

Supplementary Figure 2. Correlation between the frequency of CD137$^+$OX40$^+$ cells and the frequencies of alternative AIM$^+$ cells (OX40$^+$CD154$^+$ or CD137$^+$CD154$^+$ cells) among CD4$^+$ T cells. PBMC samples from individuals with SARS-CoV-2 infection were stimulated with OLPs of S, M, or N (1 μg/mL) for 24 hours, and the correlation between the frequency of CD137$^+$OX40$^+$ cells and the frequencies of alternative AIM$^+$ cells (OX40$^+$CD154$^+$ or CD137$^+$CD154$^+$ cells) among CD4$^+$ T cells was analyzed (n=78). Statistical analysis was performed using the two-sided Spearman correlation test.

Supplementary Figure 3

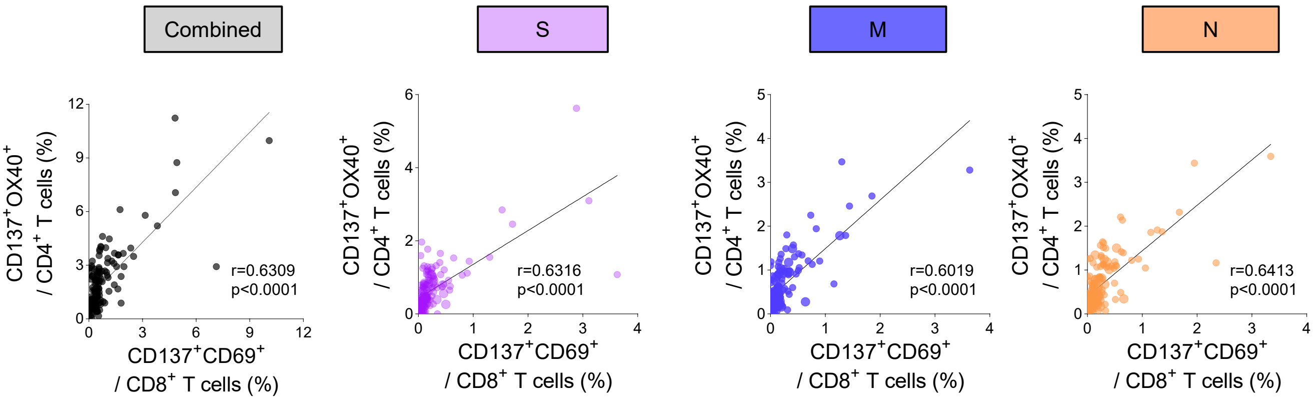

Supplementary Figure 3. Correlation of the frequency of AIM$^+$ cells between CD4$^+$ and CD8$^+$ T cells. PBMC samples from individuals with SARS-CoV-2 infection were stimulated with OLPs of S, M, or N (1 µg/mL) for 24 h and the correlation between the frequency of AIM$^+$ (CD137$^+$OX40$^+$) cells among CD4$^+$ T cells and AIM$^+$ (CD137$^+$CD69$^+$) cells among CD8$^+$ T cells was analyzed (n=146). Statistical analysis was performed using the two-sided Spearman correlation test.

Supplementary Figure 4

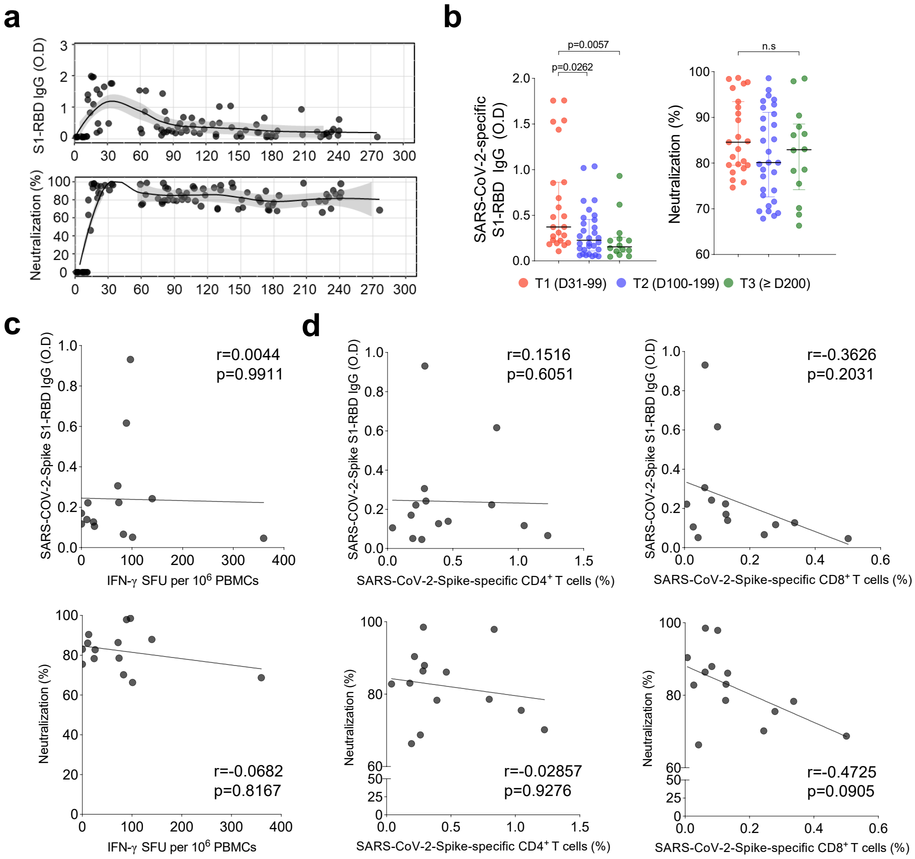

Supplementary Figure 4. Kinetics of anti-SARS-CoV-2 antibodies up to 10 months post-infection. The levels of SARS-CoV-2 S receptor binding domain (RBD)-specific IgG antibodies and SARS-CoV-2 neutralizing activity were measured in plasma samples from individuals with SARS-CoV-2 infection (n=91). **a,** Scatter plots showing the relationship between DPSO and the relative level of RBD IgG antibodies (upper) or neutralizing activity measured by SARS-CoV-2 surrogate virus neutralization assays (lower). The black line is a LOESS smooth nonparametric function, and the grey shading represents the 95% confidence interval. **b,** RBD IgG antibody levels and neutralizing activity were compared among T1 (n=23, 31 - 99 DPSO), T2 (n=30, 100 - 199 DPSO), and T3 (n=14, ≥ 200 DPSO). Data are presented as median and IQR. **c,d,** Correlation of RBD IgG antibodies and neutralizing activity with IFN-γ spot numbers (n=14) (**c**) and SARS-CoV-2 S-specific AIM^+CD4^+ and AIM^+CD8^+ T cells (n=14) (**d**). Statistical analyses were performed using the two-sided Kruskal-Wallis test with two-sided Dunns' multiple comparisons test (**b**) or two-sided Spearman correlation test (**c**,**d**). n.s., not significant.

Supplementary Figure 5

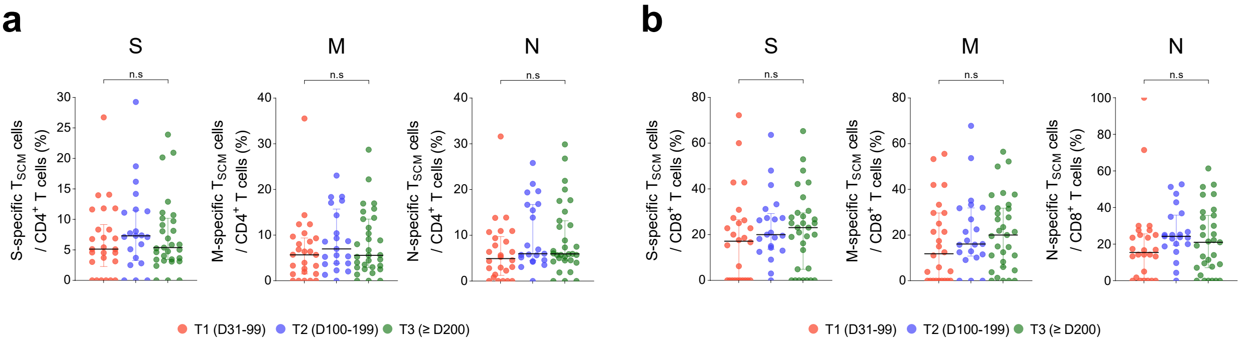

Supplementary Figure 5. Frequency of T_{SCM} cells among SARS-CoV-2-specific T cells according to days post-symptom onset. a,b, PBMC samples from COVID-19 convalescent patients were stimulated with OLPs of S, M, or N (1 μg/mL) for 24 h and the frequency of T_{SCM} ($CCR7^{+}CD45RA^{+}CD95^{+}$) cells was analyzed among AIM^{+} ($CD137^{+}OX40^{+}$) $CD4^{+}$ (**a**) and AIM^{+} ($CD137^{+}CD69^{+}$) $CD8^{+}$ T cells (**b**). The frequencies of T_{SCM} cells were compared between T1 (n=26, 31 - 99 DPSO), T2 (n=21, 100 - 199 DPSO), and T3 (n=31, ≥ 200 DPSO). Data are presented as median and IQR. Statistical analysis was performed using the two-sided Kruskal-Wallis test with Dunns' multiple comparisons test. n.s, not significant.

Supplementary Figure 6

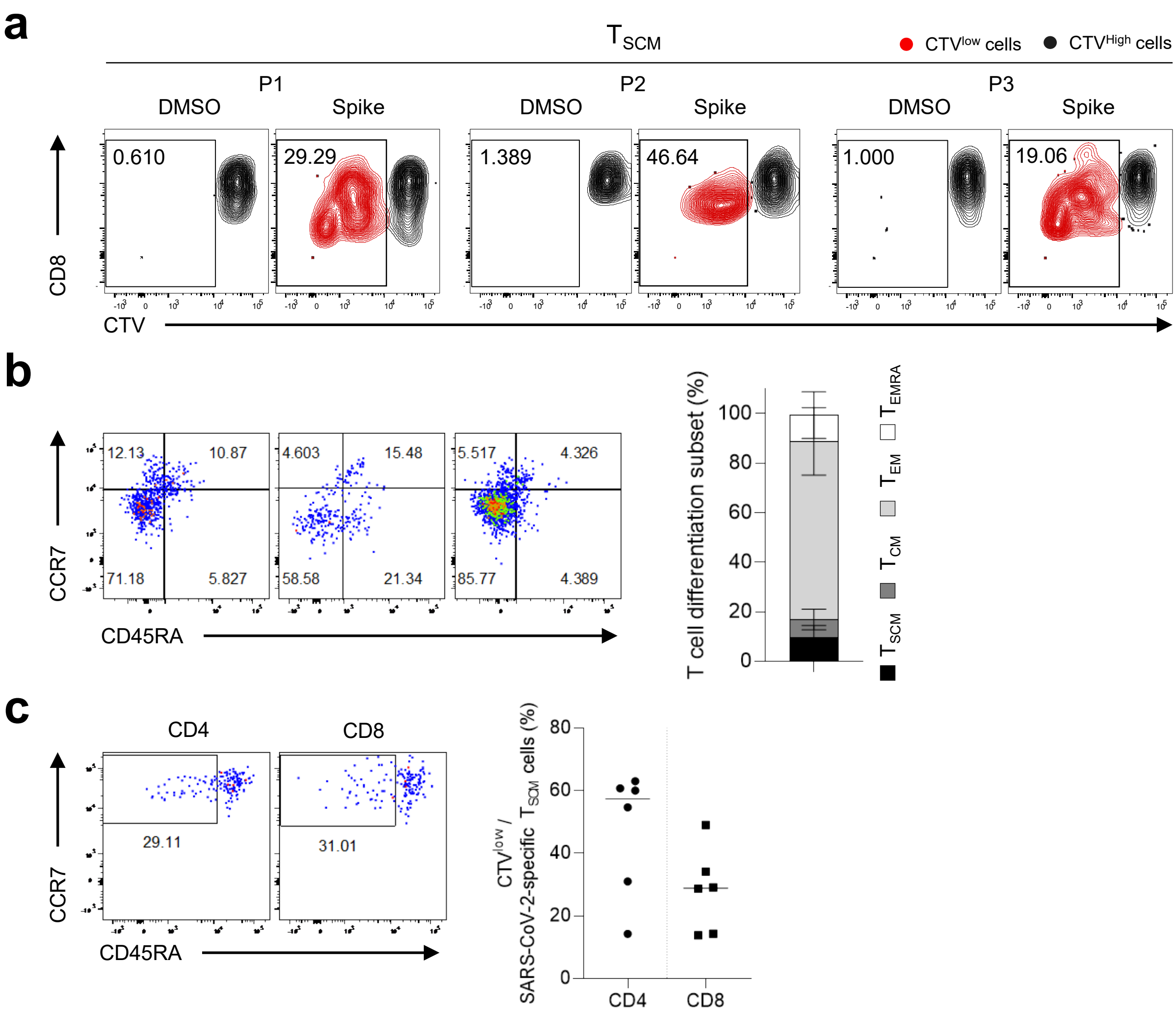

Supplementary Figure 6. Proliferation, multipotency, and self-renewal capacity of SARS-CoV-2-spike-specific T_{SCM} T cells. a, The proliferation capacity of SARS-CoV-2-specific T_{SCM} cells. Flow cytometry plots showing the proliferation of SARS-CoV-2-specific $CD8^+$ T_{SCM} cells from COVID-19 convalescent patients (n=3) following stimulation with the S OLP pool (1 μg/mL) for 7 days. **b,** Multipotency of SARS-CoV-2-specific T_{SCM} cells (n=3). Flow cytometry plot (left) and summary graph (right) showing the composition of memory subsets among the progeny of SARS-CoV-2-specific $CD8^+$ T_{SCM} cells following stimulation with the S OLP pool for 7 days. Data are presented as mean values ± SD. **c,** The self-renewal capacity of SARS-CoV-2-specific T_{SCM} cells. Flow cytometry plot (left) and summary graph (right) showing the proliferation of SARS-CoV-2-specific $CD4^+$ and $CD8^+$ T_{SCM} cells in response to IL-15 treatment (25 ng/mL) for 5 days. Following culture with IL-15, the AIM assay was performed to detect SARS-CoV-2-specific T_{SCM} cells.

Supplementary Figure 7

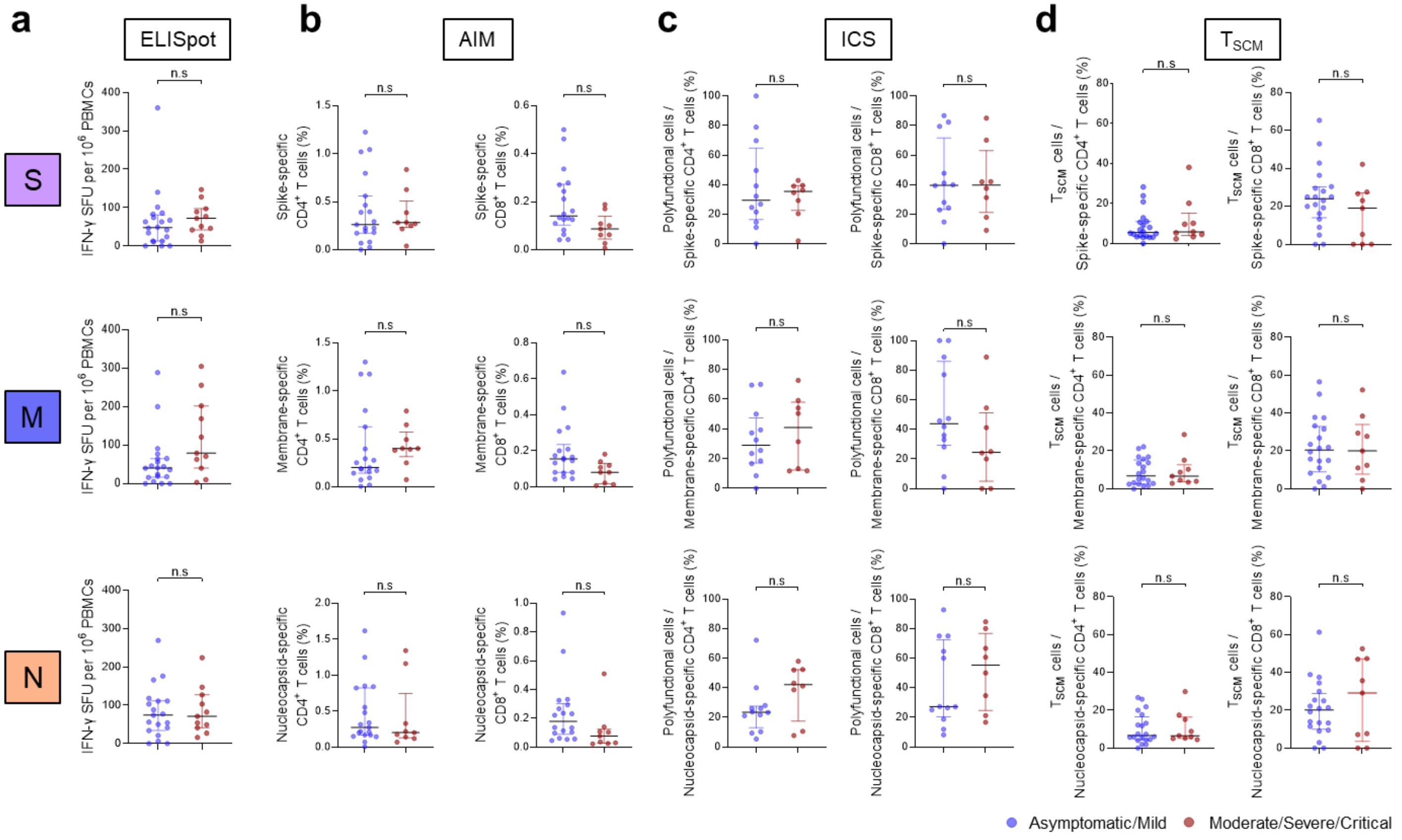

Supplementary Figure 7. Comparison of long-term SARS-CoV-2-specific T-cell responses according to peak disease severity. a-d, Long-term (≥ 200 DPSO) SARS-CoV-2-specific T-cell responses were compared between the asymptomatic/mild group (blue) and the moderate/severe/critical group (red). **a-c,** Summary graphs showing **(a)** the spot-forming units of IFN-γ-secreting cells (asymptomatic/mild group, n=20; moderate/severe/critical group, n=11), **(b)** the frequency of AIM$^+$ CD4$^+$ or CD8$^+$ T cells (asymptomatic/mild group, n=19; moderate/severe/critical group, n=10), and **(c)** the frequency of polyfunctional cells exhibiting positivity for ≥ 2 effector functions among SARS-CoV-2-specific CD4$^+$ and CD8$^+$ T cells (asymptomatic/mild group, n=12; moderate/severe/critical group, n=8). **d,** Summary graphs showing the frequency of T_{SCM} (CCR7$^+$CD45RA$^+$CD95$^+$) cells among AIM$^+$CD4$^+$ or AIM$^+$CD8$^+$ T cells (asymptomatic/mild group, n=19; moderate/severe/critical group, n=9). Data are presented as median and interquartile range (IQR). Statistical analyses were performed using the two-sided unpaired Mann-Whitney U test. n.s., not significant.

Supplementary Figure 8

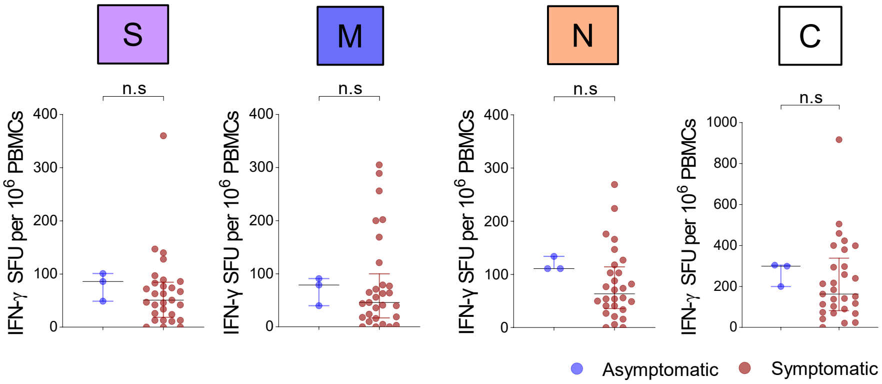

Supplementary Fig. 8. Comparison of long-term SARS-CoV-2-specific IFN-γ responses between the asymptomatic and symptomatic groups. The results of IFN-γ ELISpot assays of PBMCs from long-term COVID-19 convalescent patient samples (≥ 200 DPSO) were compared between the asymptomatic group (blue; n=3) and symptomatic group (red; n=29). Data are presented as median and IQR. Statistical analyses were performed using the two-sided unpaired Mann-Whitney U test. n.s., not significant.

Supplementary Figure 9

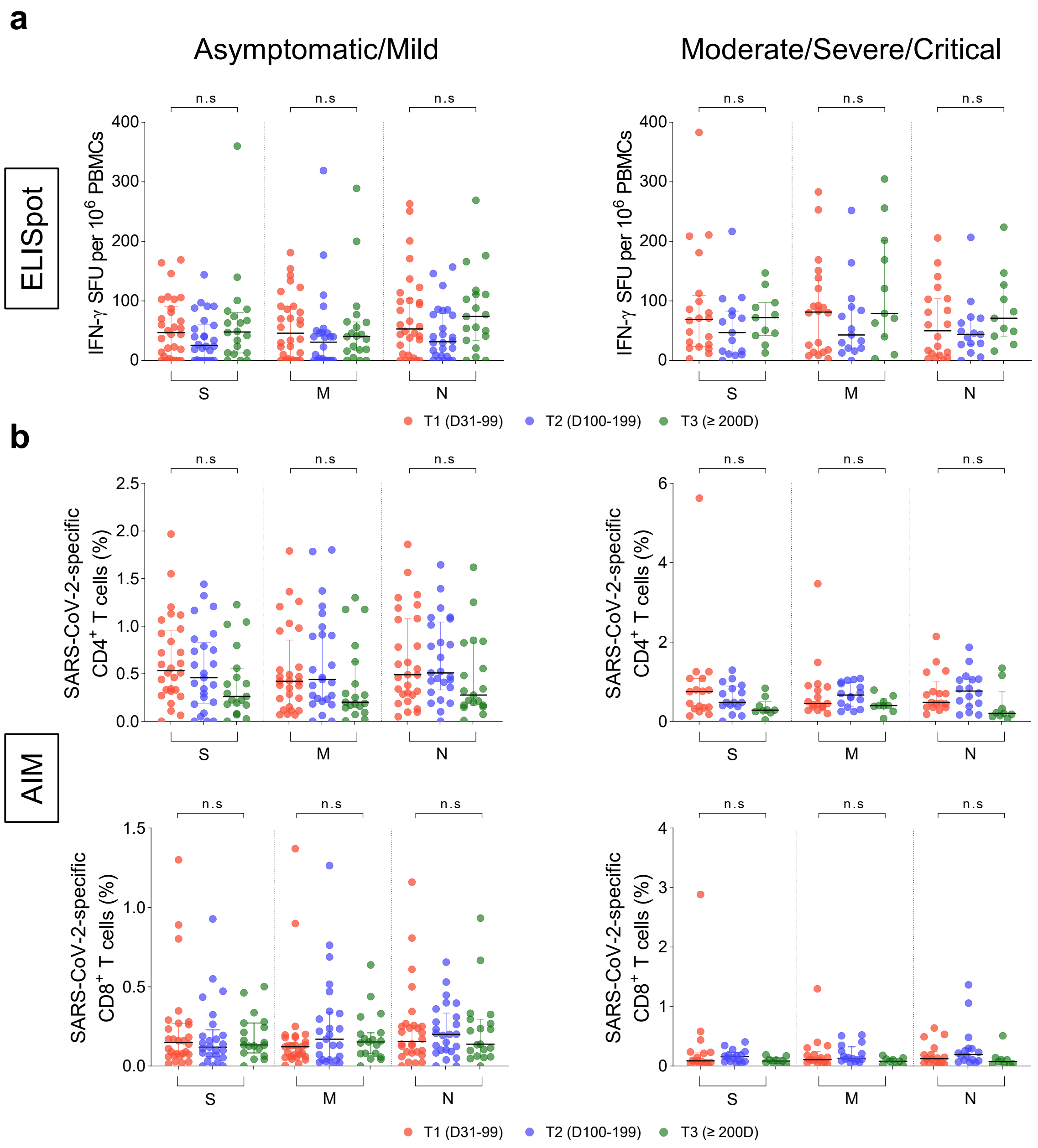

Supplementary Figure 9. Subgroup analysis of SARS-CoV-2-specific T-cell responses according to days post-symptom onset. Within the asymptomatic/mild group or moderate/severe/critical group, the spot-forming units of IFN-γ-secreting cells (**a**) or the frequencies of SARS-CoV-2-specific AIM$^+$CD4$^+$ T cells and AIM$^+$CD8$^+$ T cells (**b**) were compared among T1 (31 - 99 DPSO), T2 (100 - 199 DPSO), and T3 (≥ 200 DPSO). **a,** The magnitude of IFN-γ responses in the asymptomatic/mild group (n=30 (T1), n=26 (T2), and n=20 (T3)), and the moderate/severe/critical group (n=19 (T1), n=15 (T2), and n=11 (T3)). **b,** The frequency of SARS-CoV-2-specific AIM$^+$ CD4$^+$ (upper) and CD8$^+$ (lower) T cells in the asymptomatic/mild group (n=28 (T1), n=25 (T2), and n=19 (T3)), and the moderate/severe/critical group (n=17 (T1), n=16 (T2), and n=9 (T3)). Data are presented as median and IQR. Statistical analyses were performed using the two-sided Kruskal-Wallis test with two-sided Dunns' multiple comparisons test. n.s., not significant.

Supplementary Figure 10

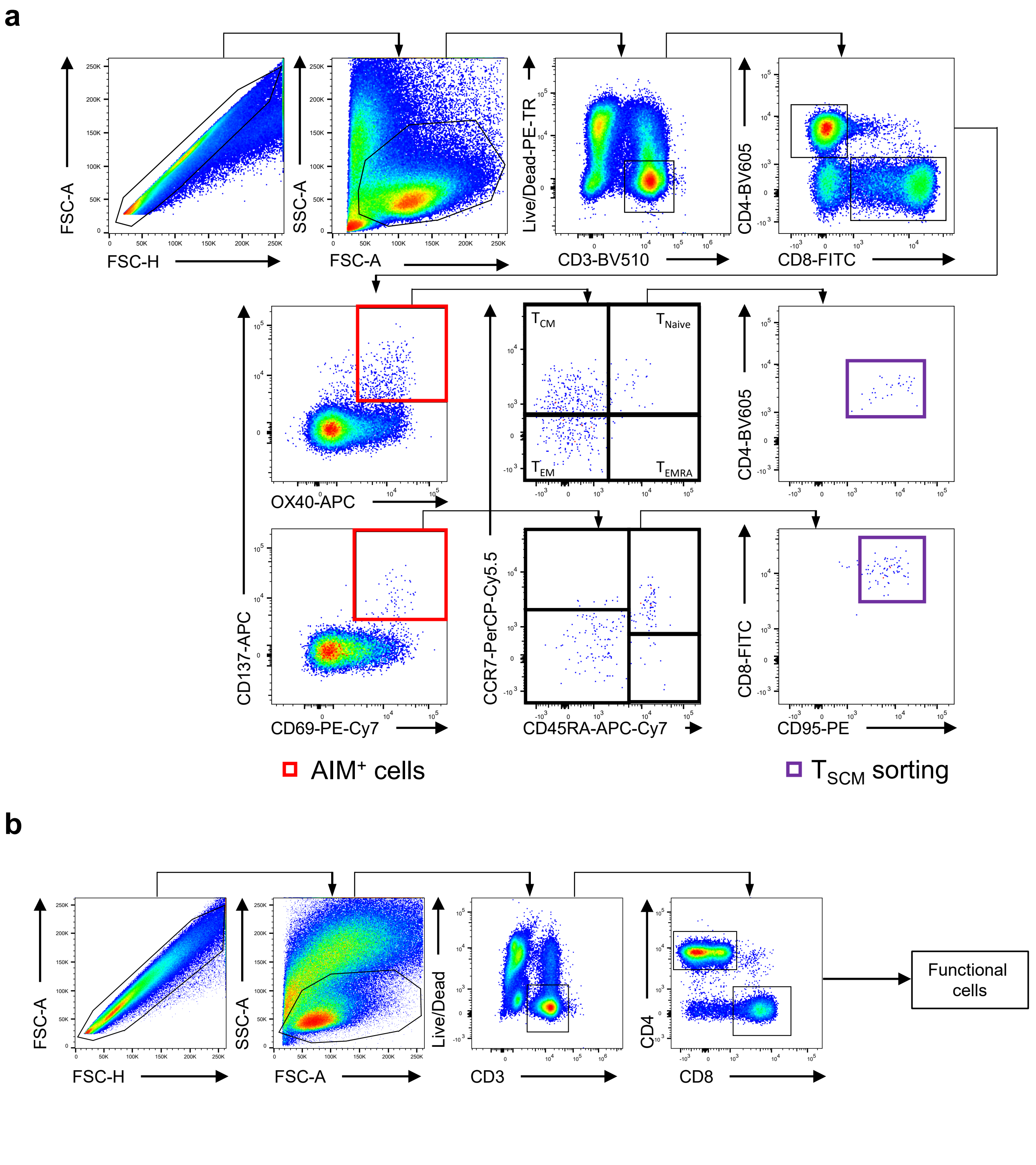

Supplementary Figure 10. Gating strategies for cell sorting and the detection of AIM+ cells and cytokine-producing cells. a, Gating strategy to examine the frequencies of AIM+ (CD137+OX40+) CD4+ or AIM+ (CD137+CD69+) CD8+ T cells and to sort T_{SCM} cells (AIM+CCR7+CD45RA+CD95+) from PBMC samples from individuals with SARS-CoV-2 infection presented on Figure 2,3 and 5e. **b,** Gating strategy to examine the frequencies of functional cells (IFN-γ+, IL-2+, or CD107a+) from PBMC samples from individuals with SARS-CoV-2 infection presented on figure 5.

Supplementary Table 1

Supplementary Table 1. Characteristics of enrolled patients

Parameter	COVID-19 (n=101)
Age (years)	19-96 (median 39, IQR 30)
Gender	
Female (%)	57 (56.4%)
Male (%)	44 (43.6%)
Ethnicity	
Korean	99 (98.0%)
Non-Korean	2 (2.0%)
Peak disease severity[a]	
Asymptomatic, n	7 (6.9%)
Mild, n	46 (45.5%)
Moderate, n	25 (24.8%)
Severe, n	14 (13.9%)
Critical, n	9 (8.9%)
DPSO at sample collection	1-317 (median 94, IQR 117)
Blood collection	
Multiple time points, n	56
2	29
3	18
4	9
Single time point, n	45
Assays	Total 193 samples
IFN-γ ELISpot assays	153 samples (n=87)
ICS	90 samples (n=52)
AIM assays	146 samples (n=80)
MHC-I multimer staining	15 samples (n=11)
Proliferation assays	18 samples (n=18)
ELISA	91 samples (n=66)
T_{SCM} polyfunctionality assays	8 samples (n=8)
T_{SCM} proliferation assays	3 samples (n=3)

IQR, interquartile range; DPSO, days post-symptom onset; ELISpot, enzyme-linked immunospot; ICS, intracellular cytokine staining; AIM, activation-induced marker; ELISA, enzyme-linked immunosorbent assay; T_{SCM}, stem cell-like memory T cell.

[a]Disease severity was defined by the NIH severity of illness categories.

Supplementary Table 2

Supplementary Table 2. Sample composition in each assay according to disease severity and days post-symptom onset.

Sample distribution	ELISpot			AIM			ICS		
	T1[a] (n=49)	T2[b] (n=41)	T3[c] (n=31)	T1 (n=45)	T2 (n=41)	T3 (n=28)	T1 (n=27)	T2 (n=32)	T3 (n=22)
Asymptomatic	0/49 (0%)	3/41 (7.3%)	2/31 (6.5%)	0/45 (0%)	2/41 (4.9%)	1/28 (3.6%)	0/27 (0%)	3/32 (9.4%)	2/22 (9.1%)
Mild	30/49 (61.2%)	23/41 (56.1%)	18/31 (58.1%)	28/45 (62.2%)	23/41 (56.1%)	18/28 (64.3%)	17/27 (63.0%)	21/32 (65.6%)	12/22 (54.5%)
Moderate	11/49 (22.4%)	8/41 (19.5%)	6/31 (19.4%)	9/45 (20.0%)	9/41 (22.0%)	5/28 (17.9%)	7/27 (25.9%)	5/32 (15.6%)	5/22 (22.7%)
Severe	5/49 (10.2%)	4/41 (9.5%)	4/31 (12.9%)	5/45 (11.1%)	4/41 (9.5%)	3/28 (10.7%)	3/27 (11.1%)	2/32 (6.3%)	2/22 (9.1%)
Critical	3/49 (6.1%)	3/41 (7.3%)	1/31 (3.2%)	3/45 (6.7%)	3/41 (7.3%)	1/28 (3.6%)	0/27 (0%)	1/32 (3.1%)	1/22 (4.5%)

ELISpot, enzyme-linked immunospot; AIM, Activation-induced marker; ICS, Intracellular cytokine staining.
[a]T1, 31 - 99 DPSO; [b]T2, 100 - 199 DPSO; [c]T3, ≥ 200 DPSO.

Supplementary Table 3

Supplementary Table 3. Flow cytometry reagents

REAGENT	SOURCE	IDENTIFIER	Dilution
FITC Anti-human CD107a (clone H4A3)	BD Biosciences	#555800	1:100
APC Anti-human CD137 (clone 4B4-1)	BD Biosciences	#550890	1:100
BV421 Anti-human CD137 (clone 4B4-1)	BD Biosciences	#564091	1:100
PE-CF594 Anti-human CD14 (clone MϕP9)	BD Biosciences	#562335	1:100
APC Anti-human CD154 (clone TRAP1)	BD Biosciences	#555702	1:100
PE-CF594 Anti-human CD19 (clone HIB19)	BD Biosciences	#562294	1:100
BV510 Anti-human CD27 (clone L128)	BD Biosciences	#563092	1:100
BV510 Anti-human CD3 (clone UCHT1)	BD Biosciences	#563109	1:100
BV786 Anti-human CD3 (clone UCHT1)	BD Biosciences	#565491	1:100
BV605 Anti-human CD4 (clone RPA-T4)	BD Biosciences	#562658	1:100
FITC Anti-human CD4 (clone RPA-T4)	BD Biosciences	#555346	1:100
PerCP™Cy5.5 Anti-human CD4 (clone RPA-T4)	BD Biosciences	#560650	1:100
AF700 Anti-human TNF (clone Mab11)	BD Biosciences	#557996	1:100
BB515 Anti-human CD45RO (clone UCHL1)	BD Biosciences	#564529	1:100
PE-Cy7 Anti-human CD69 (clone FN50)	BD Biosciences	#557745	1:100
APC-Cy7 Anti-human CD8 (clone SK1)	BD Biosciences	#560179	1:100
BV605 Anti-human CD8 (clone SK1)	BD Biosciences	#564116	1:100
BV711 Anti-human CD8 (clone RPA-T8)	BD Biosciences	#563677	1:100
PE Anti-human CD95 (clone DX2)	BD Biosciences	#555674	1:100
PE-Cy7 Anti-human IFN-γ (clone 4S.B3)	BD Biosciences	#557844	1:100
APC Anti-human IL-2 (clone MQ1-17H12)	BD Biosciences	#554567	1:100
BV786 Anti-human Ki-67 (clone B56)	BD Biosciences	#563756	1:100
PerCP™Cy5.5 Anti-human CCR7 (clone G043H7)	BioLegend	#353220	1:100
PE Anti-human CD137 (clone 4B4-1)	BioLegend	#309804	1:100
APC Anti-human CD3 (clone HIT3a)	BioLegend	#300312	1:100
APC-Cy7 Anti-human CD45RA (clone HI100)	BioLegend	#304128	1:100
FITC Anti-human CD8 (clone RPA-T8)	BioLegend	#301050	1:100
BV421 Anti-human OX40 (clone Ber-ACT35)	BioLegend	#350014	1:100
BV421 Anti-human PD-1 (clone EH12.2H7)	BioLegend	#329920	1:100
PE-Cy7 Anti-human TIGIT (clone MBSA43)	Invitrogen	#25-9500-42	1:100
APC YLQPRTFLL (SARS-CoV-2 S_{269}) HLA-A*0201 Pentamer	Proimmune	#4339	1:20
APC GILGFVFTL (IAV MP_{58}) HLA-A*0201 Dextramer	Immudex	#WB2161	1:20
APC NLVPMVATV (CMV $pp65_{495}$) HLA-A*0201 Dextramer	Immudex	#WB2132	1:20
