# Supplementary material for: SARS-CoV-2-specific T cell memory is sustained in COVID-19 convalescent patients for 10 months with successful development of stem cell-like memory T cells
Source: Nat Commun. 2021 Jun 30;12:4043. doi: 10.1038/s41467-021-24377-1 (PMC8245549; doi:10.1038/s41467-021-24377-1)
Supplement: Supplementary file 3 — Reporting Summary [file 41467_2021_24377_MOESM3_ESM.pdf]

Corresponding author(s): Eui-Cheol Shin

Last updated by author(s): Jun 11, 2021

Reporting Summary

Nature Research wishes to improve the reproducibility of the work that we publish. This form provides structure for consistency and transparency in reporting. For further information on Nature Research policies, see our Editorial Policies and the Editorial Policy Checklist.

Statistics

For all statistical analyses, confirm that the following items are present in the figure legend, table legend, main text, or Methods section.

n/a	Confirmed	
☐	☒	The exact sample size (n) for each experimental group/condition, given as a discrete number and unit of measurement
☐	☒	A statement on whether measurements were taken from distinct samples or whether the same sample was measured repeatedly
☐	☒	The statistical test(s) used AND whether they are one- or two-sided<br>*Only common tests should be described solely by name; describe more complex techniques in the Methods section.*
☒	☐	A description of all covariates tested
☐	☒	A description of any assumptions or corrections, such as tests of normality and adjustment for multiple comparisons
☐	☒	A full description of the statistical parameters including central tendency (e.g. means) or other basic estimates (e.g. regression coefficient) AND variation (e.g. standard deviation) or associated estimates of uncertainty (e.g. confidence intervals)
☐	☒	For null hypothesis testing, the test statistic (e.g. F, t, r) with confidence intervals, effect sizes, degrees of freedom and P value noted<br>*Give P values as exact values whenever suitable.*
☒	☐	For Bayesian analysis, information on the choice of priors and Markov chain Monte Carlo settings
☒	☐	For hierarchical and complex designs, identification of the appropriate level for tests and full reporting of outcomes
☒	☐	Estimates of effect sizes (e.g. Cohen's d, Pearson's r), indicating how they were calculated

Our web collection on statistics for biologists contains articles on many of the points above.

Software and code

Policy information about availability of computer code

Data collection	For ELISpot assays, data were collected with AID ELISpot v.7.0.<br>For flow cytometry, data were collected with BD FACSDIVA v.9.0.<br>For ELISA, data were collected with Biotek Gen5.
Data analysis	Flow cytometry<br>Data from flow cytometry were analyzed with FlowJo™v.10.7.1 software for MAC.<br>Data regarding polyfunctionality of T cells were analyzed with SPICE v.6.1.<br><br>Statistical analysis<br>Data were analyzed with GraphPad Prism software v.9.0.<br>For local regression (Loess) analysis, R v.4.0.2 with packages of dplyr v.0.8.5, tidyr v.3.0.1, ggplot2 v.3.3.1, tidyverse v.1.3.0, ggthemes v.0.5.1, and svglite v.1.2.3.2.

For manuscripts utilizing custom algorithms or software that are central to the research but not yet described in published literature, software must be made available to editors and reviewers. We strongly encourage code deposition in a community repository (e.g. GitHub). See the Nature Research guidelines for submitting code & software for further information.

Data

Policy information about availability of data

All manuscripts must include a data availability statement. This statement should provide the following information, where applicable:

- Accession codes, unique identifiers, or web links for publicly available datasets
- A list of figures that have associated raw data
- A description of any restrictions on data availability

All data supporting the findings of this study are available within the main manuscript and the supplementary files or provided upon reasonable request. Raw data corresponding to all the main and supplementary figures are included in the Source Data file. Source data are provided with this paper.

Field-specific reporting

Please select the one below that is the best fit for your research. If you are not sure, read the appropriate sections before making your selection.

- [x] Life sciences
- [ ] Behavioural & social sciences
- [ ] Ecological, evolutionary & environmental sciences

For a reference copy of the document with all sections, see nature.com/documents/nr-reporting-summary-flat.pdf

Life sciences study design

All studies must disclose on these points even when the disclosure is negative.

Sample size	We recruited 101 individuals with SARS-CoV-2 infection and 8 individuals without SARS-CoV-2 infection. Sample sizes were based on the maximum number of patient samples where days post-symptom onset and peak disease severity at the time of symptom onset were available. Unexposed individual samples from pre-pandemic period were randomly selected to match the gender and age of individuals with SARS-CoV-2 infection.
Data exclusions	For ELISpot assays, if negative control wells had >40 SFU/10^6 peripheral blood mononuclear cells (PBMCs), the results were excluded from analysis (Peng et al, Nat Immunol 2020).<br>For activation-induced marker (AIM) analysis, if the percentage of AIM+(OX40+CD137+)CD4+ or AIM+(CD69+CD137+)CD8+ T cells after stimulation with SARS-CoV-2 overlapping peptide pools were equal or lower than AIM+ cells of corresponding DMSO controls, data were excluded from phenotypic analysis (Grifoni et al, Cell 2020).
Replication	Experiments did not include replicates as all participants and their data are unique.
Randomization	Participants were allocated based on the days post-symptom onset and peak disease severity at the time of symptom onset. Covariates including age (mean 39; 19-96) and gender (male to female ratio = 56:44) did not differ significantly between experimental groups.
Blinding	Investigators were blinded to the days post-symptom onset and peak disease severity during data collection of ELISpot, AIM, ELISA, multimer, and intracellular staining (ICS) assays. For cell sorting and proliferation assays, investigators were blinded to the gender and age but not days post-symptom onset to determine the long-term maintenance of SARS-CoV-2-TSCM cells.

Reporting for specific materials, systems and methods

We require information from authors about some types of materials, experimental systems and methods used in many studies. Here, indicate whether each material, system or method listed is relevant to your study. If you are not sure if a list item applies to your research, read the appropriate section before selecting a response.

Materials & experimental systems

n/a	Involved in the study	
[ ]	[x]	Antibodies
[x]	[ ]	Eukaryotic cell lines
[x]	[ ]	Palaeontology and archaeology
[x]	[ ]	Animals and other organisms
[ ]	[x]	Human research participants
[x]	[ ]	Clinical data
[x]	[ ]	Dual use research of concern

Methods

n/a	Involved in the study	
[x]	[ ]	ChIP-seq
[ ]	[x]	Flow cytometry
[x]	[ ]	MRI-based neuroimaging

Antibodies

Antibodies used	ELISpot<br><br>Mabtech

Anti-human IFN-γ monoclonal coating antibody - 1-D1K - 3420-3-250 - RRID:AB_223578 (Dilution 1:1000)
Anti-human IFN-γ monoclonal capture antibody, biotinylated - 7-B6-1 - 3420-6-250 - RRID:AB_907273 (Dilution 1:1000)

Flow cytometry

BD Biosciences
Anti-human CD107a - FITC - H4A3 - Cat#555800 - RRID:AB_11024655
Anti-human CD137 - APC - 4B4-1 - Cat#550890 - RRID:AB_398477
Anti-human CD137 - BV421 - 4B4-1 - Cat#564091 - RRID:AB_2722503
Anti-human CD14 - PE-CF594 - MϕP9 - Cat#562335 - RRID:AB_11153663
Anti-human CD154 - APC - TRAP1 - Cat#555702 - RRID:AB_398610
Anti-human CD19 - PE-CF594 - HIB19 - Cat#562294 - RRID:AB_11154408
Anti-human CD27 - BV510 - L128 - Cat#563092 - RRID:AB_2313577
Anti-human CD3 - BV510 - UCHT1 - Cat#563109 - RRID:AB_2732053
Anti-human CD3 - BV786 - UCHT1 - Cat#565491 - RRID:AB_2739260
Anti-human CD4 - BV605 - RPA-T4 - Cat#562658 - RRID:AB_2744420
Anti-human CD4 - FITC - RPA-T4 - Cat#555346 - RRID:AB_395751
Anti-human CD4 - PerCP™Cy5.5 - RPA-T4 - Cat#560650 - RRID:AB_1727476
Anti-human CD45RO - BB515 - UCHL1 - Cat#564529 - RRID:AB_2744408
Anti-human CD69 - PE-Cy7 - FN50 - Cat#557745 - RRID:AB_396851
Anti-human CD8 - APC-Cy7 - SK1 - Cat#560179 - RRID:AB_1645481
Anti-human CD8 - BV605 - SK1 - Cat#564116 - RRID:AB_2869551
Anti-human CD8 - BV711 - RPA-T8 - Cat#563677 - RRID:AB_2744463
Anti-human CD95 - PE - DX2 - Cat#555674 - RRID:AB_396027
Anti-human IFN-γ - PE-Cy7 - 4S.B3 - Cat#557844 - RRID:AB_396894
Anti-human IL-2 - APC - MQ1-17H12 - Cat#554567 - RRID:AB_398571
Anti-human Ki-67 - BV786 - B56 - Cat#563756 - RRID:AB_2732007
Anti-human TNF - AF700 - Mab11 - Cat#557996 - RRID:AB_396978

BioLegend
Anti-human CCR7 - PerCP™Cy5.5 - G043H7 - Cat#353220 - RRID:AB_10916121
Anti-human CD137 - PE - 4B4-1 - Cat#309804 - RRID:AB_314783
Anti-human CD3 - APC - HIT3a - Cat#300312 - RRID:AB_314048
Anti-human CD45RA - APC-Cy7 - HI100 - Cat#304128 - RRID:AB_10708880
Anti-human CD8 - FITC - RPA-T8 - Cat#301050 - RRID:AB_2562055
Anti-human OX40 - BV421 - Ber-ACT35 - Cat#350014 - RRID:AB_2564184
Anti-human PD-1 - BV421 - EH12.2H7 - Cat#329920 - RRID:AB_10960742

Invitrogen
Anti-human TIGIT - PE-Cy7 - MBSA43 - Cat#25-9500-42 - RRID:AB_2573548

Immudex
Anti-human GILGFVFTL (IAV MP58) HLA-A*0201 Dextramer - APC - NA - Cat#WB2161 - N/A
Anti-human NLVPMVATV (CMV pp65495) HLA-A*0201 Dextramer - APC - NA - Cat#WB2132 - N/A

Proimmune
Anti-human YLQPRTFLL (SARS-CoV-2 S269) HLA-A*0201 Pentamer - APC - NA - Cat#4339 - N/A

Validation

ELISpot

Mabtech
Anti-human IFN-γ mAb - https://www.mabtech.com/products/anti-human-ifn-gamma-antibody-1-d1k-purified-3420-3
Anti-human IFN-γ mAb, biotinylated - https://www.mabtech.com/products/anti-human-ifn-gamma-antibody-7-b6-1-biotinylated-3420-6

Flow cytometry

BD Biosciences
Anti-human CD107a - FITC - https://www.bdbiosciences.com/kr/applications/research/intracellular-flow/intracellular-antibodies-and-isotype-controls/anti-human-antibodies/fitc-mouse-anti-human-cd107a-h4a3/p/555800
Anti-human CD137 - APC - https://www.bdbiosciences.com/kr/applications/research/t-cell-immunology/regulatory-t-cells/surface-markers/human/apc-mouse-anti-human-cd137-4b4-1/p/550890
Anti-human CD137 - BV421 - https://www.bdbiosciences.com/kr/reagents/research/antibodies-buffers/immunology-reagents/anti-human-antibodies/cell-surface-antigens/bv421-mouse-anti-human-cd137-4b4-1/p/564091
Anti-human CD14 - PE-CF594 - https://www.bdbiosciences.com/kr/applications/research/stem-cell-research/hematopoietic-stem-cell-markers/human/negative-markers/pe-cf594-mouse-anti-human-cd14-mp9-also-known-as-mp-9/p/562335
Anti-human CD154 - APC - https://www.bdbiosciences.com/kr/applications/research/t-cell-immunology/regulatory-t-cells/surface-markers/human/apc-mouse-anti-human-cd154-trap1-also-known-as-trap-1/p/555702

Anti-human CD19 - PE-CF594 - https://www.bdbiosciences.com/kr/applications/research/clinical-research/oncology-research/blood-cell-disorders/surface-markers/human/pe-cf594-mouse-anti-human-cd19-hib19/p/562294
Anti-human CD27 - BV510 - https://www.bdbiosciences.com/kr/applications/research/clinical-research/oncology-research/blood-cell-disorders/surface-markers/human/bv510-mouse-anti-human-cd27-l128/p/563092
Anti-human CD3 - BV510 - https://www.bdbiosciences.com/us/applications/research/t-cell-immunology/th-1-cells/surface-markers/human/bv510-mouse-anti-human-cd3-ucht1-also-known-as-ucht-1-ucht-1/p/563109
Anti-human CD3 - BV786 - https://www.bdbiosciences.com/kr/reagents/research/antibodies-buffers/immunology-reagents/anti-human-antibodies/cell-surface-antigens/bv786-mouse-anti-human-cd3-ucht1-also-known-as-ucht-1-ucht-1/p/565491
Anti-human CD4 - BV605 - https://www.bdbiosciences.com/kr/applications/research/t-cell-immunology/th-1-cells/surface-markers/human/bv605-mouse-anti-human-cd4-rpa-t4/p/562658
Anti-human CD4 - FITC - https://www.bdbiosciences.com/kr/applications/research/t-cell-immunology/th-1-cells/surface-markers/human/fitc-mouse-anti-human-cd4-rpa-t4/p/555346
Anti-human CD4 - PerCP™Cy5.5 - https://www.bdbiosciences.com/kr/applications/research/stem-cell-research/hematopoietic-stem-cell-markers/human/negative-markers/percp-cy55-mouse-anti-human-cd4-rpa-t4/p/560650
Anti-human CD45RO - BB515 - https://www.bdbiosciences.com/kr/applications/research/t-cell-immunology/regulatory-t-cells/surface-markers/human/bb515-mouse-anti-human-cd45ro-uchl1/p/564529
Anti-human CD69 - PE-Cy7 - https://www.bdbiosciences.com/kr/applications/research/t-cell-immunology/regulatory-t-cells/surface-markers/human/pe-cy7-mouse-anti-human-cd69-fn50-also-known-as-fn-50/p/557745
Anti-human CD8 - APC-Cy7 - https://www.bdbiosciences.com/kr/reagents/research/antibodies-buffers/immunology-reagents/anti-human-antibodies/cell-surface-antigens/apc-h7-mouse-anti-human-cd8-sk1/p/560179
Anti-human CD8 - BV605 - https://www.bdbiosciences.com/kr/reagents/research/antibodies-buffers/immunology-reagents/anti-human-antibodies/cell-surface-antigens/bv605-mouse-anti-human-cd8-sk1/p/564116
Anti-human CD8 - BV711 - https://www.bdbiosciences.com/kr/reagents/research/antibodies-buffers/immunology-reagents/anti-human-antibodies/cell-surface-antigens/bv711-mouse-anti-human-cd8-rpa-t8/p/563677
Anti-human CD95 - PE - https://www.bdbiosciences.com/eu/applications/research/t-cell-immunology/regulatory-t-cells/surface-markers/human/pe-mouse-anti-human-cd95-dx2/p/555674
Anti-human IFNγ - PE-Cy7 - https://www.bdbiosciences.com/kr/applications/research/t-cell-immunology/th-1-cells/intracellular-markers/cytokines-and-chemokines/human/pe-cy7-mouse-anti-human-ifn--4sb3/p/557844
Anti-human IL-2 - APC - https://www.bdbiosciences.com/kr/applications/research/t-cell-immunology/th-1-cells/intracellular-markers/cytokines-and-chemokines/human/apc-rat-anti-human-il-2-mq1-17h12/p/554567
Anti-human Ki-67 - BV786 - https://www.bdbiosciences.com/kr/applications/research/intracellular-flow/intracellular-antibodies-and-isotype-controls/anti-human-antibodies/bv786-mouse-anti-ki-67-b56/p/563756
Anti-human TNF - AF700 - https://www.bdbiosciences.com/us/applications/research/t-cell-immunology/th-1-cells/intracellular-markers/cytokines-and-chemokines/human/alexa-fluor-700-mouse-anti-human-tnf-mab11/p/557996

BioLegend
Anti-human CCR7 - PerCP™Cy5.5 - https://www.biolegend.com/en-us/products/percp-cyanine5-5-anti-human-cd197-ccr7-antibody-7539
Anti-human CD137 - PE - https://www.biolegend.com/en-us/products/pe-anti-human-cd137-4-1bb-antibody-1510
Anti-human CD3 - APC - https://www.biolegend.com/en-us/products/apc-anti-human-cd3-antibody-749
Anti-human CD45RA - APC-Cy7 - https://www.biolegend.com/en-us/products/apc-cyanine7-anti-human-cd45ra-antibody-7056
Anti-human CD8 - FITC - https://www.biolegend.com/en-us/products/fitc-anti-human-cd8a-antibody-834
Anti-human OX40 - BV421 - https://www.biolegend.com/en-us/products/brilliant-violet-421-anti-human-cd134-ox40-antibody-7335
Anti-human PD-1 - BV421 - https://www.biolegend.com/en-us/products/brilliant-violet-421-anti-human-cd279-pd-1-antibody-7191

Invitrogen
Anti-human TIGIT - PE-Cy7 - https://www.thermofisher.com/antibody/product/TIGIT-Antibody-clone-MBSA43-Monoclonal/25-9500-42

Immudex
Anti-human GILGFVFTL (IAV MP58) HLA-A*0201 Dextramer - https://www.iedb.org/epitope/20354
Anti-human NLVPMVATV (CMV pp65495) HLA-A*0201 Dextramer - https://www.iedb.org/epitope/44920

Proimmune
Anti-human YLQPRTFLL (SARS-CoV-2 S269) HLA-A*0201 Pentamer -
Peng, Y., Mentzer, A. J., Liu, G., Yao, X., Yin, Z., Dong, D., ... & López-Camacho, C. (2020). Broad and strong memory CD4+ and CD8+ T cells induced by SARS-CoV-2 in UK convalescent individuals following COVID-19. Nature immunology, 21(11), 1336-1345.
Rha, M. S., Jeong, H. W., Ko, J. H., Choi, S. J., Seo, I. H., Lee, J. S., ... & Kim, J. H. (2020). PD-1-expressing SARS-CoV-2-specific CD8+ T Cells Are Not Exhausted, but Functional in Patients with COVID-19. Immunity.

Human research participants

Policy information about studies involving human research participants

Population characteristics	We recruited 101 individuals with SARS-CoV-2 infection and 8 individuals without SARS-CoV-2 infection. The peak disease severity was evaluated according to the NIH severity of illness categories: asymptomatic (n=7), mild (n=46), moderate (n=25), severe (n=14), and critical (n=9). Whole blood samples were obtained longitudinally (2-4 time points) from 56 patients or at a single time point from 45 patients. Whole blood was collected 1 to 317 days post-symptom onset (DPSO). Finally, a total of 193 PBMC samples were analyzed. Among 193 PBMC samples, 37 samples were obtained in the acute phase when viral RNA was still detected (1 - 33 DPSO), and 156 samples were obtained in the convalescent phase after the negative conversion of viral RNA (31 - 317 DPSO). The demographic and clinical characteristics of enrolled patients are presented in Supplementary Table 1.
Recruitment	This study recruited 101 PCR-confirmed SARS-CoV-2 infected patients admitted to Chungbuk National University Hospital and Korea University Ansan Hospital between February 2020 and February 2021. Patients were admitted according to same admission criteria in two hospitals to avoid any potential bias. Unexposed donors were recruited randomly during pre-pandemic period.
Ethics oversight	This study was reviewed and approved by the institutional review board of Chungbuk National University Hospital (2020-03-036-001) and Korea University Ansan Hospital (2020AS0122) and conducted according to the principles of the Declaration of Helsinki. Informed consents were obtained from all donors and patients.

Note that full information on the approval of the study protocol must also be provided in the manuscript.

Flow Cytometry

Plots

Confirm that:

- [x] The axis labels state the marker and fluorochrome used (e.g. CD4-FITC).
- [x] The axis scales are clearly visible. Include numbers along axes only for bottom left plot of group (a 'group' is an analysis of identical markers).
- [x] All plots are contour plots with outliers or pseudocolor plots.
- [x] A numerical value for number of cells or percentage (with statistics) is provided.

Methodology

Sample preparation	Plasma samples were isolated from peripheral blood after centrifugation. After centrifugation, PBMCs were isolated by density gradient centrifugation using Lymphocyte Separation Medium (Corning). After isolation, the cells were cryopreserved in fetal bovine serum (FBS; Corning) with 10% dimethyl sulfoxide (Sigma Aldrich). Cryopreserved PBMCs were thawed at 37°C waterbath and were added with DNase for 5 minutes at room temperature. Cells were washed with RPMI 1640 + 10% FBS + 1% penicillin and streptomycin. After washing, cells were resuspended in appropriate medium for further experiments.
Instrument	Data were collected using BD FACS LSR II.
Software	Data from flow cytometry were analyzed with FlowJo™v.10.7.1 software for MAC.
Cell population abundance	Cell population abundance was reported as a proportion of a specific population (% of CD4+, % of CD8+, % of AIM+CD4+, % of AIM+CD8+, etc)
Gating strategy	For all the experiments, single cells were gated on a forward scatter height vs. forward scatter area plot. Lymphocytes were gated on forward vs. side scatter plot based on their size and granularity. To exclude dead cells and non-T cells, LIVE/DEAD Fixable Dead Cell reactive dye+, CD14+, and CD19+ cells were gated out. T cells were gated with CD3, CD4, and CD8 gating.<br>For AIM+ assays, AIM+CD4+ or AIM+CD8+ cells were gated according to the corresponding DMSO controls. Subsequently, AIM+ cells were gated with CCR7, CD45RA, and CD95 for analysis of differentiation state.<br>For multimer staining, antigen-specific cells were gated based negative cells following live CD3+ T cell gating. Subsequently, multimer+ cells were gated with CCR7, CD45RA, and CD95 for analysis of differentiation state.<br>For ICS assays, IFN-γ+, IL-2+, TNF+, and CD107a+ cells were gated according to the corresponding DMSO controls.<br>For proliferation assays, CTVlow cells and Ki-67+ cells were gated according to the corresponding DMSO controls.<br>For stem cell-like memory T cell proliferation assays, CTVlow cells were gated according to the corresponding DMSO controls.

- [x] Tick this box to confirm that a figure exemplifying the gating strategy is provided in the Supplementary Information.
