# Supplementary material for: SARS-CoV-2-specific T cell memory is sustained in COVID-19 convalescent patients for 10 months with successful development of stem cell-like memory T cells
Source: Nat Commun. 2021 Jun 30;12:4043. doi: 10.1038/s41467-021-24377-1 (PMC8245549; doi:10.1038/s41467-021-24377-1)
Supplement: Supplementary file 4 — Description of Additional Supplementary Files [file 41467_2021_24377_MOESM4_ESM.pdf]

Description of Additional Supplementary Files

File Name: Supplementary Data 1

Description: Clinical and sampling details of the blood samples used in each figure. Data includes patient number, sample serial number, days post-symptom onset, disease severity, age, and sex.
